# Alternative Approaches to Characterizing Disparate Care by Race, Ethnicity, and Insurance Between Hospitals

**DOI:** 10.3390/ijerph22101514

**Published:** 2025-10-02

**Authors:** Alina Kung, Yingtong Chen, Bian Liu, Louisa W. Holaday, Karen McKendrick, Albert L. Siu

**Affiliations:** 1 Brookdale Department of Geriatrics and Palliative Medicine, Icahn School of Medicine at Mount Sinai, New York, NY 10029, USA; yingtong.chen@mssm.edu (Y.C.); karen.mckendrick@mssm.edu (K.M.); albert.siu@mssm.edu (A.L.S.); 2 Department of Population Health Science and Policy, Icahn School of Medicine at Mount Sinai, New York, NY 10029, USA; bian.liu@mountsinai.org; 3 Division of General Internal Medicine, Icahn School of Medicine at Mount Sinai, New York, NY 10029, USA; louisa.holaday@mountsinai.org; 4 Institute for Health Equity Research, Icahn School of Medicine at Mount Sinai, New York, NY 10029, USA; 5 Geriatric Research, Education, and Clinical Center, James J. Peters VA Medical Center, Bronx, New York, NY 10468, USA

**Keywords:** hospitals, Medicare, Medicaid, racial groups, ethnicity, minority health, socioeconomic disparities in health, healthcare disparities

## Abstract

Identifying hospitals that disproportionately serve minority and publicly insured patients is important because patients at these hospitals often experience worse outcomes. Studies commonly identify disproportion by using the top decile of hospitals with the greatest proportion of Black discharges nationally. Our study aimed to identify a broader measure that accounts for disproportion by multiple characteristics. Using fee-for-service Medicare data, we classified hospitals as either serving disproportionately or not, examined overlaps in classification, and assessed differences in hospital quality. We found that using a combined measure for any hospitals in the top decile or above a threshold of twice their local healthcare market average of Black, Hispanic, minority, or dual-eligible discharges classified 28.1% (*n* = 680/2420) of hospitals as serving disproportionately, compared to only 10% (*n* = 242/2420) when using the top decile of a single characteristic. The combined measure detected moderate differences in hospital star quality ratings (mean difference of 0.57–0.87, all *p*-values < 0.001; standardized mean difference: 0.50–0.79, 95% CIs all above 0). The combined measure identified hospitals that were smaller, more rural, and served other minorities, namely, Asian and American Indian populations. Future work should consider using this combined measure to more comprehensively identify hospitals that disproportionately serve minority or publicly insured patients.

## 1. Introduction

Despite historic efforts towards reducing disparities in hospital care [1,2,3,4,5], disparate hospital care associated with race and insurance remains significant [6] and consequential [7]. Disparate care, which we define as the extent to which individuals of different groups occupy or experience differently resourced health environments [8], is the outcome of structural, economic, cultural, interpersonal, and intrapersonal factors operating at multiple levels [9]. Research suggests that these separate conditions in combination with uneven outcomes are unhealthy for patients and society [10,11]. In addition to being associated with worse health outcomes [10,11], disparate conditions exacerbate system dynamics such as cycles of underinvestment, capital and workforce deficiencies, and decreased healthcare access for communities [12,13,14,15,16,17,18,19,20].

Disparate care can occur in association with multiple and overlapping characteristics. Hospitals that disproportionately serve Black or minority patients are associated with lower funding [12,13], lower quality [7], and poorer outcomes, often for Black and White patients alike [21,22,23,24,25,26,27,28]. Meanwhile, hospitals that disproportionately serve low-income, underinsured, or uninsured patients, commonly referred to as safety-net hospitals, show similar patterns [29,30,31]. Safety-net hospitals are located in more deprived areas, have fewer resources, see sicker and more psychosocially complex patients, and consequently are of lower quality and have poorer health outcomes [29,30,31]. There is high overlap between non-White (minority) race/ethnicity and low socioeconomic status (SES) among patients [32] and therefore also high overlap between hospitals that disproportionately serve low-income patients and hospitals that disproportionately serve minority patients [31]. Few studies have examined how disparate hospital care associated with race/ethnicity intersects with disparate hospital care associated with SES, although recent exceptions exist [6,33].

Existing conceptual frameworks acknowledge how unevenness in the distributions of hospital patients by race/ethnicity interacts with socioeconomic factors to produce disparities in hospital outcomes [7,34]. However, both sets of authors were primarily interested in how hospitals disproportionately served patients of certain racial/ethnic groups and did not consider race/ethnicity and SES as measures of disproportion simultaneously. Our study’s theoretical contribution addresses this gap. We draw on the Andersen Model of Health Service Use [35], which describes how predisposing (e.g., race/ethnicity) and enabling (e.g., health insurance) factors at multiple levels affect health exposures and behaviors that ultimately affect health outcomes. We add social determinants [36,37] to the Andersen model to consider disparities associated with race/ethnicity simultaneously with disparities associated with SES (proxied by dual-eligible status). While the Index of Concentration at the Extremes achieves a similar goal [38,39], our contribution is in our focus on and the applicability of our model to hospital populations.

Interest in measuring disparate care related to differences in the racial, ethnic, and insurance composition of patient populations has been increasing. While distributions of patients by race, ethnicity, and insurance have multiple features [8,40], researchers most commonly identify areas with these composition differences using *evenness* and identify hospitals with these composition differences using *disproportion* [41,42,43]. Evenness of areas is typically measured through the Dissimilarity Index (DI), which can be interpreted as the proportion of group X that would need to switch subunits in order to have even populations across subunits [44]. Disproportion for hospitals is most commonly measured through the top decile of the greatest proportion of discharges for a group of interest nationally [23,26,45,46,47,48,49]. The characteristic historically studied is Black race, given the legacy of harmful policies and practices towards Black populations in the United States [50,51]. However, hospitals can disproportionately serve patients with other characteristics, including other races, ethnicities, income, or insurance.

Being able to identify health facilities as disproportionately serving certain populations by multiple features, such as race/ethnicity and insurance, has implications for policy and practice. By knowing where and how individuals occupy or experience different environments, health leaders and policy makers could design interventions that decrease the concentration of minority and low-income patients in poorly resourced and lower-quality hospitals and target additional resources and quality-improvement initiatives in these hospitals. While challenging, this may ensure more even distributions of patients, resources, and quality across hospitals and consequently improve health outcomes for all. Identifying the presence and extent of uneven racial/ethnic and insurance compositions of patients in relation to outcomes could also be utilized as a structural measure of hospitals. This measure could play a role in informed patient choices, reimbursement methodologies, and efficiency [52]. Identifying hospitals that disproportionately serve certain populations ideally should occur in a reliable and intuitively understood way.

Measuring disparate care in relation to variation in the racial/ethnic and insurance composition of hospitalized populations is challenging on numerous fronts. For validity, there is variability regarding which feature is of greatest interest, such as evenness, exposure, or disproportion relative to an aggregated group [41,42]. For reliability, measures vary in the area size and boundaries used; formulas for evenness, exposure, or disproportion; and threshold values defining facilities as disproportionately serving certain populations [42,43]. For accuracy, it is unclear which “true” or gold-standard measure would serve as the best comparison.

Several developments in research and practice suggest that a measure of disproportion relative to the local community, in addition to relative to a national sample, is needed. Hebert et al. conducted a simulation study and found that the traditional minority-serving approach (the top decile relative to a national sample) underestimated the true effect of between-hospital disparities in quality on outcomes [53]. Their finding suggests that a measure of disproportion that captures a greater number of hospitals that still detects known differences in quality may more accurately describe disparities in hospital outcomes [53]. Moreover, in 2022, authors from the US News and World Report called for purposeful measures to track and close disparities in hospital outcomes [54]. They specifically discussed the importance of analyzing the extent to which hospital populations reflect the demographics of Medicare beneficiaries residing in the surrounding community and factoring this information into hospital rankings [54]. A first step to addressing these gaps and challenges would be to test specific definitions and thresholds, characterize the hospitals disproportionately serving certain populations using these measures, and ensure their ability to detect known differences in outcomes.

Our study identifies hospitals using the most common measure of hospital disproportion (the top decile of the greatest proportion of discharges for a group of interest) [23,46,55] and compares this approach against a proposed measure of serving above twice the local healthcare market average for a group of interest. We also test whether using a combined measure, consisting of all hospitals disproportionately serving certain populations by *either* the top decile *or* the proposed measure, across multiple characteristics of Black race, Hispanic ethnicity, non-White (minority) race, or dual-eligible status, detects known differences in hospital quality. We use data on fee-for-service (FFS) Medicare discharges from the Centers for Medicare and Medicaid Services (CMS) and Dartmouth Atlas to classify hospitals as disproportionately serving certain populations or not and perform *t*-tests to assess differences in hospital quality. We hypothesize that using the combined measure, beyond the top decile for a single group of interest, will allow for the identification of additional hospitals disproportionately serving certain populations while still capturing meaningful quality differences between hospitals.

## 2. Materials and Methods

### 2.1. Data and Sample

Data sources for hospital characteristics included cross-sectional data of fee-for-service Medicare beneficiaries from the 2023 CMS Medicare Inpatient Hospitals Provider file (hereafter: Provider file) [56] and 2023 CMS hospital star quality ratings, which were reported in the 2025 archived file [57,58]. Hospital characteristics included proportions of Black, Hispanic, non-White (minority), and dual-eligible beneficiaries discharged that year; rurality; census region (Northeast, Midwest, South, or West); number of FFS beneficiaries discharged per year; and CMS star quality rating. The rurality of hospitals was designated using United States Department of Agriculture Rural–Urban Commuting Area (RUCA) codes that range from 1 to 10, with 1–3 considered urban and to 4–10 considered rural [59].

Data on area characteristics were sourced and derived from the Dartmouth Atlas of Healthcare [60]. The area, or geographic unit of analysis, we chose was the hospital referral region (HRR). An HRR represents the local healthcare market for tertiary medical care and is widely used in health services research [60]. HRR characteristics included the number of hospitals in the HRR and the evenness of the Black, Hispanic, non-White (minority), and dual-eligible FFS Medicare patient distributions between hospitals, as measured through the Dissimilarity Index (DI). We linked hospitals to their HRRs using the Dartmouth Atlas 2019 HRR-ZIP crosswalk file [61], which contained the most recent data available.

We identified our analytic sample through multiple steps. We first identified eligible hospitals (*n* = 3093) using the 2023 CMS Provider file. The discharge data in the Provider file are restricted to short-term hospitalizations for the FFS population, which were the focus of our study; long-term care, critical access hospitals, rehabilitation, and psychiatric discharges are not included [62]. Because the Dissimilarity Index becomes unreliable at smaller sample sizes [63,64], we excluded hospitals with fewer than 250 fee-for-service Medicare discharges (*n* = 535). We also excluded hospitals that were missing quality star ratings (*n* = 138), which can occur when hospitals do not participate in reporting or when there is insufficient data [57]. Our final sample included 2420 hospitals spanning all 306 HRRs.

### 2.2. Measures

#### 2.2.1. Areas with Uneven Racial/Ethnic and Insurance Patient Compositions—Dissimilarity Index

We used the Dissimilarity Index (DI) as our measure of evenness in patient racial/ethnic and insurance compositions in an area. We have described the calculation and equation for the DI in a prior paper [6]. In brief, the DI can be interpreted as the proportion of FFS beneficiaries of a group of interest that would need to switch hospitals in an HRR in order for the hospitals to have even proportions of that group across hospitals. For example, the HRR for Boston, MA, has 37 hospitals. The DI for Hispanic beneficiaries in Boston is 0.277; thus, 27.7% of the Hispanic FFS Medicare population would need to switch hospitals for the 37 hospitals to have the same proportion of Hispanic beneficiaries across them.

#### 2.2.2. Hospital Disproportion—Top Decile and 2×HRR

To classify hospitals as disproportionately serving certain populations (hereafter: disproportionate hospitals) or not, we used two measures. The first measure was the top decile of the proportion of discharges of a group of interest when ranked nationally [23,46,55]. We first calculated the percentage of FFS discharges that were Black, Hispanic, non-White (minority), or dual-eligible for each hospital. We then ranked the 2420 hospitals from the greatest to the lowest proportion of discharges for each group and created an indicator variable for hospitals in the top decile. The top decile thresholds were as follows: 23.1% Black discharges, 17.9% Hispanic discharges, 48.2% minority discharges, and 45.7% dual-eligible discharges.

The second measure classified hospitals as disproportionate if they served more than twice the HRR mean proportion of a group of interest (hereafter: 2×HRR). The advantage of this measure is how it accounts for regional variations that may be missed when using national rankings. We chose 2×HRR after testing various cut-offs, including 1.5, 2, and 2.5 times the HRR mean (Table A1). We found that 2.5×HRR may be an overly high threshold, as it classified even fewer hospitals as disproportionate than the top decile, even when summing multiple characteristics, including Black-disproportionate, Hispanic-disproportionate, minority-disproportionate, and dual-disproportionate hospitals (7.1%, *n* = 171/2420 hospitals). Alternately, 1.5×HRR was insufficiently specific (32.8%, *n* = 794/2420 hospitals) (Table A1). In sensitivity analyses (Table A2), 1.5×HRR detected only small, rather than moderate, quality differences between hospitals.

To calculate 2×HRR, we first summed the number of FFS beneficiaries in the HRR. Then we calculated the mean percentage of FFS beneficiaries that were Black, Hispanic, non-White (minority), or dual-eligible, separately, for all the hospitals in the HRR. If the hospital percentage of discharges was at least twice the HRR mean percentage, a hospital was considered disproportionate by the 2×HRR measure for the characteristic of interest. For example, in the Boston, MA, HRR, Boston Medical Center (BMC) had 10.9% Hispanic discharges in 2023 compared to the HRR average of 3.8% Hispanic discharges. Because BMC’s percentage of Hispanic discharges exceeds 7.6%, or twice the HRR average, it serves a disproportionate number of Hispanic beneficiaries in its area. BMC would not be considered to serve disproportionately by the top decile Hispanic measure, as it did not meet the threshold of 17.9% or more Hispanic discharges.

**Figure 1:**
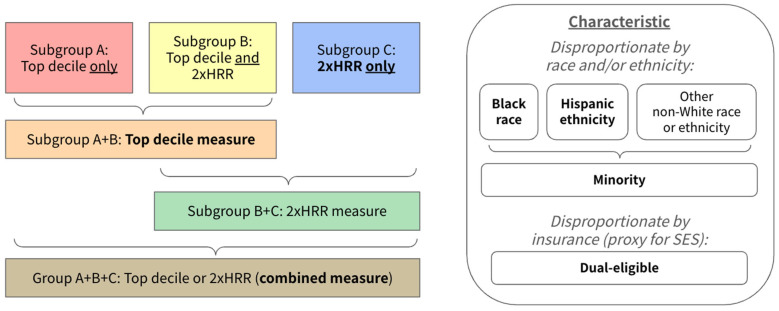
Groupings, subgroupings, and characteristics for measures of disproportion. **Bolded** groups indicate foci of analysis for this study.

These two measures, the top decile and 2×HRR, were applied for the characteristics of Black race, Hispanic ethnicity, non-White (minority) race/ethnicity, and dual-eligible insurance status. We included the minority group, which aggregates non-White groups, including American Indian/Alaska Native, Asian and Pacific Islander, Black, Hispanic, and other groups, because this minority category allows the study of non-White groups that might be too small in number to consider separately (Figure 1, right panel). Dual-eligible refers to being dually eligible for Medicare and Medicaid, the public insurance program covering low-income populations. Dual-eligible thus describes disproportion by insurance and also serves as a proxy for low SES.

Hospitals that disproportionately served certain populations were in one of three mutually exclusive subgroups—classified as disproportionate by the top decile measure only (subgroup A), by both the top decile and 2×HRR measures (subgroup B), or by the 2×HRR measure only (subgroup C) (Figure 1, left panel). Because prior work has utilized the top decile as a threshold measure for disproportion [23,46,47,55], we focused on the inclusive top decile measure (subgroup A + B) and compared this against the incremental hospitals identified as disproportionate when using the 2×HRR only measure (subgroup C) (Figure 1, left panel). Finally, we also utilized the combination of subgroups A + B + C, which identified hospitals that were disproportionate either by the top decile *or* the 2×HRR measure (hereafter: combined measure) (Figure 1, left panel).

#### 2.2.3. Hospital Quality

To characterize hospital quality, we used CMS star quality ratings [57]. These ratings range from 1 to 5 and are updated annually, with typically a 1–3-year delay between data release and the actual reporting period; thus, the 2025 file was used for 2023 ratings [58]. The ratings summarize a variety of measures across five areas of quality in a single star rating for each hospital [57]. A hospital summary score is calculated for each hospital by taking the weighted average of the hospital’s scores for each measure group, which include mortality, safety, readmission, patient experience, and timely and effective care [57].

### 2.3. Statistical Analysis

We calculated descriptive statistics, including counts and proportions, for the various measures of disproportion. For variables with normally distributed data, we report means and standard deviations (SDs) and the results of *t*-tests. For variables where the data were not normally distributed, we report medians and interquartile ranges (IQRs) and use non-parametric tests. Specifically, we performed Mann–Whitney U tests to assess differences in baseline hospital and area characteristics between varying measures of disproportion and to assess differences in hospital quality between disproportionate and non-disproportionate groups. In addition to *t*-tests, we report quality differences by the mean difference and standardized mean difference (SMD) using Cohen’s d [65,66]. By convention, we consider SMD values of 0.2–0.5 a small difference, 0.51–0.8 a medium difference, and >0.8 a large difference and interpret a confidence interval that lies entirely above 0 as statistically significant [65]. Analyses were performed between April and June 2025 using SAS 9.4 [67] and STATA 18.5 [68]. Full data will be made available upon request. This study did not involve human subjects and was therefore exempt from IRB review.

## 3. Results

**Figure 2:**
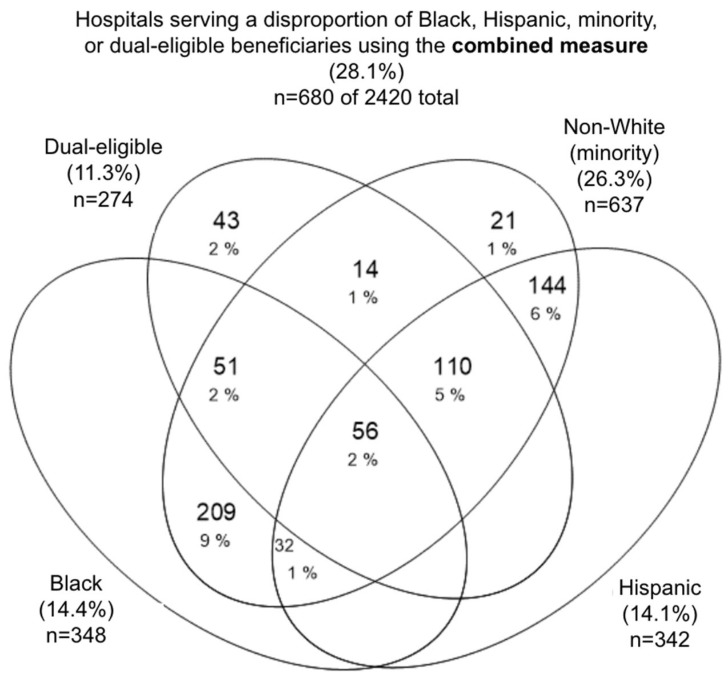
Overlap in number and proportion of hospitals disproportionately serving Black, Hispanic, any minority (inclusive of Black, Hispanic, and other non-White beneficiaries), and dual-eligible beneficiaries by the top decile or 2×HRR measure.

Our initial sample of 3093 hospitals was narrowed to a final analytic sample of 2420 hospitals, as described in Section 2.1. The hospitals spanned 306 HRRs. The average racial, ethnic, and dual-eligible compositions of hospital discharges were as follows: 78.7% White, 21.3% non-White (minority) (which is inclusive of 9.3% Black and 6.5% Hispanic), and 27.5% dual-eligible. The median number of FFS discharges was 1496 per year (interquartile range (IQR) 25–75%: 772–2759), and 22.4% of hospitals were in rural areas. HRRs had a median number of 12 hospitals (IQR: 6–21). After excluding 15 HRRs that only had one hospital and thus could not have DIs calculated for them, the average HRR DIs were 0.249 for Black, 0.171 for Hispanic, 0.197 for minority, and 0.158 for dual-eligible FFS Medicare beneficiaries. Over one quarter (28.1%, *n* = 680 of 2420) of hospitals were classified as minority-, Black-, Hispanic-, or dual-disproportionate by the combined measure of either the top decile or 2×HRR (Figure 2). The remaining hospitals (*n* = 1740, 71.9%) did not disproportionately serve minority, Black, Hispanic, or dual-eligible populations by the combined measure.

### 3.1. The Number and Proportion of Hospitals Disproportionately Serving Certain Populations Varies Greatly Based on Measure

#### 3.1.1. Top Decile Measure

**Figure A1:**
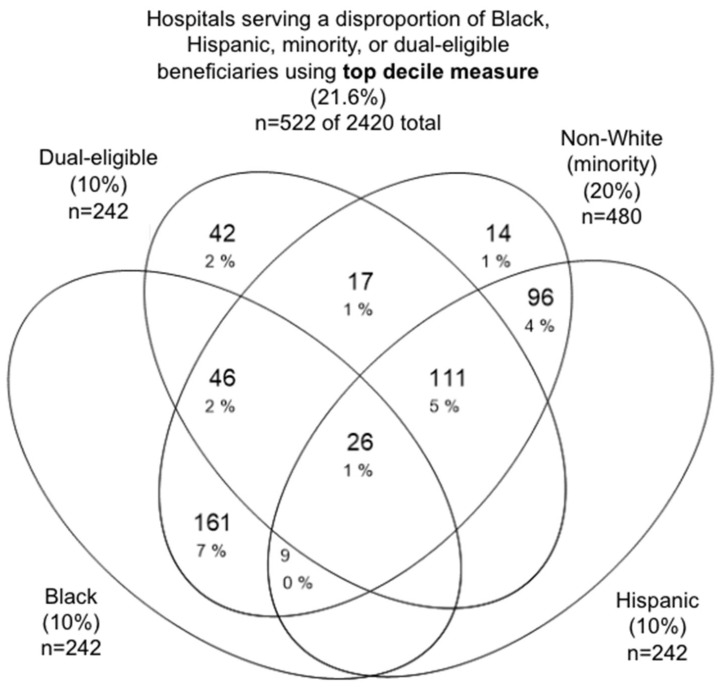
Number and proportion of hospitals disproportionately serving Black, Hispanic, minority, or dual-eligible beneficiaries using **top decile measure**.

To be in the top decile, the thresholds the hospitals had to exceed were 23.2% Black, 17.9% Hispanic, 48.3% minority, and 45.7% dual-eligible discharges. By definition, the top decile measure identified 10% of the sample of hospitals (*n* = 242) as serving a disproportionate number of a particular group. If the disproportion measure were expanded to include hospitals that met *any* top decile group, specifically Black, Hispanic, minority, *or* dual-eligible FFS discharges, roughly over a fifth of hospitals would be considered disproportionate (21.6%, *n* = 522/2420) (Figure A1). While Black, Hispanic, minority, and dual-eligible populations often cluster in metropolitan areas [31], only 26 hospitals were in the top decile for Black, Hispanic, minority, *and* dual-eligible discharges (Figure A1).

#### 3.1.2. 2×HRR Only Measure

**Figure A2:**
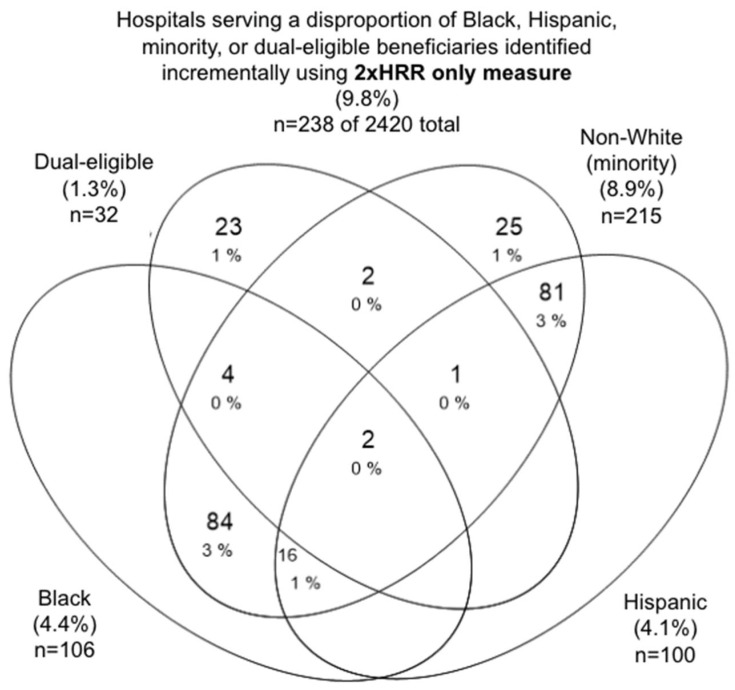
Number and proportion of hospitals disproportionately serving Black, Hispanic, minority, or dual-eligible beneficiaries using the **2×HRR only measure**.

**Figure A3:**
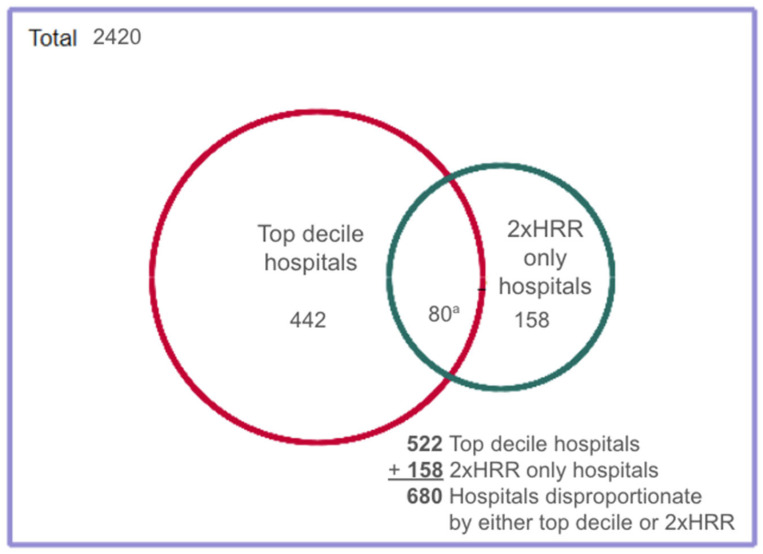
Overlap between top decile and 2×HRR only measures for hospitals disproportionately serving Black, Hispanic, minority, *or* dual-eligible beneficiaries. ^a^ Overlap in top decile and 2×HRR only is possible due to depicting multiple characteristics. For example, a hospital that disproportionately serves beneficiaries by 2×HRR may be in the top decile of hospitals that serve dual beneficiaries. However, top decile and 2×HRR only remain mutually exclusive categories when considered by one characteristic.

Hospitals met the 2×HRR measure if they served equal to or greater than two times their HRR mean percentage discharges for the group of interest. This mean percentage varied based on the composition of FFS beneficiaries in each HRR. Hospitals that exceeded a median of 21.4% Black (IQR: 9.2–41.3%), 9.1% Hispanic (IQR: 2.0–24.0%), 36.0% minority (IQR: 15.9–60.7%), or 49.8% dual (IQR: 40.0–65.1%) discharges were classified as 2×HRR (subgroup B + C in Figure 1) (Table A1). Using the 2×HRR only measure identified an additional 106 (4.4%), 100 (4.1%), 215 (8.9%), and 32 (1.3%) hospitals as serving a disproportionate number of Black, Hispanic, minority, or dual-eligible beneficiaries, respectively (Figure A2). Only four hospitals met the 2×HRR threshold for minority, Black, Hispanic, *and* dual-eligible populations (Figure A2). In total, the 2×HRR measure detected an additional 158 hospitals that were not included when using the top decile definition (Figure A3).

#### 3.1.3. Combined Measure

When using the combined measure of the top decile *or* 2×HRR, we found that 680 hospitals, or 28.1% of the total sample (*n* = 2420), served a disproportionate number of Black, Hispanic, minority, or dual-eligible FFS beneficiaries (Table 1, Figure A3). Minority-disproportionate hospitals included hospitals that were Black-disproportionate, Hispanic-disproportionate, or disproportionate for another non-White group. Using the minority-disproportionate category identified an additional 35 hospitals that would otherwise not be considered disproportionate by Black, Hispanic, or dual-eligible status (Table A3, Figure 2). These hospitals largely had 2×HRR means of Asian/Pacific Islander or American Indian/Alaska Native populations (Table A3).

We also examined the overlap in measures of disproportion between race/ethnicity and dual-eligible status. We found that 30.7% (107/348), 48.5% (166/342), and 36.2% (231/637), respectively, of the Black-serving, Hispanic-serving, and minority-serving hospitals also disproportionately served dual-eligible beneficiaries (Figure 2). Only 15.7% (43/274) of hospitals that were dual-serving were not also minority-serving (Figure 2).

### 3.2. Hospital and Area Characteristics Vary Significantly Between Top Decile and 2×HRR Only Groups

To determine whether hospitals incrementally identified with the 2×HRR only group differed from those identified with the more commonly used measure of the top decile, we compared their hospital and area characteristics. We found that hospitals additionally captured by the 2×HRR only measure tended to be in more rural areas, have smaller volumes, be in HRRs with slightly more evenly distributed populations (lower DIs), and be of higher quality compared to the top decile group (Table 2).

### 3.3. Hospital and Area Characteristics Vary Significantly Between Disproportionate and Non-Disproportionate Hospitals

We used *t*-tests and Mann–Whitney U tests to examine whether mean hospital and area characteristics between hospitals that served certain populations disproportionately and those that did not differed significantly. Patterns between these two groups of hospitals were consistent whether identified by the top decile measure (Table 3) or the combined measure (Table 4). Disproportionate hospitals had fewer discharges per year and were in HRRs with a greater number of hospitals and greater unevenness in their distribution of Black, Hispanic, minority, and dual-eligible beneficiaries (higher DI) (Table 3 and Table 4). A greater proportion of disproportionate hospitals were in urban areas compared to hospitals that were not disproportionate.

### 3.4. Both the Top Decile and the Combined Measure Detected Significant Quality Differences

Most notably, we found that disproportionate hospitals on average were of lower quality compared to hospitals that were not disproportionate. The greatest quality differences were detected with the top decile measure, with mean differences of −0.73, −0.65, −0.67, and −0.89 stars for Black-serving, Hispanic-serving, minority-serving, and dual-serving hospitals, respectively (Table 3). Standardized mean differences (SMDs) for the top decile exceeded 0.5, indicating a moderate, significant difference (Table A4, all 95% CIs > 0). When using the combined measure to identify disproportionate hospitals, differences in quality were slightly attenuated in comparison to the top decile measure. Mean differences were −0.57, −0.58, −0.60, and −0.87 stars for Black-serving, Hispanic-serving, minority-serving, and dual-serving hospitals, respectively (Table 4), and SMDs remained greater than 0.5 across groups, indicating a persistent significant difference (Table A4, all 95% CIs > 0). When using the 2×HRR measure (subgroup B + C in Figure 1) alone to identify disproportionate hospitals, differences in quality were statistically significant, albeit smaller (SMDs of −0.44, −0.43, −0.42, and −0.74 for Black-serving, Hispanic-serving, minority-serving, and dual-serving hospitals, respectively) (Table A5).

## 4. Discussion

Our study found that while using the top decile measure for a single characteristic (10%, *n* = 242) detected significant quality differences between disproportionate and non-disproportionate hospitals, a combined measure using multiple characteristics identified a larger sample (*n* = 680) while still detecting significant quality differences. Using 2×HRR, a newly proposed measure, where hospitals exceed twice the local healthcare market (HRR) mean proportion of discharges for a certain group, we detected an additional 138 hospitals as disproportionate that were not detected by the top decile. These additional hospitals had fewer discharges per year, were in HRRs with more evenly distributed hospital populations by race and dual-eligible status, were more likely to be in rural areas, and included hospitals that disproportionately served other non-White groups, including Asian and American Indian populations.

While prior studies have focused on disproportion in hospitals for Black–White populations, there are numerous advantages to classifying hospitals as disproportionate using multiple characteristics simultaneously. Firstly, disproportions associated with different characteristics may share common causes and produce similar outcomes, as underscored by the differences in star quality ratings in our study. Secondly, identifying a larger sample of hospitals as disproportionate can increase the statistical power of tests, leading to more accurate, reliable, and precise results. Thirdly, an intersectional approach may better reflect the lived realities of patients, who experience differences in hospital quality related to a variety of social factors.

Our study contributes several insights into how disproportions in hospital populations associated with race, ethnicity, and insurance compare and intersect. While prior work has shown patterns of lower quality in Black-serving hospitals [46,69,70], we found that the magnitude of these quality differences is slightly greater for Hispanic-serving and minority-serving hospitals and noticeably greater for dual-serving hospitals (Section 3.4). This finding suggests the continued importance of SES, proxied by public insurance, in disparate hospital outcomes. Furthermore, when examining the overlap between disproportions associated with race/ethnicity and dual-eligible status, we found that nearly half of Hispanic-serving hospitals were also dual-serving hospitals and that less than a fifth of dual-serving hospitals were not minority-serving (Figure 2). The close relationship between minority and dual-eligible status has also been demonstrated in other studies examining compositions of hospitalized patients [6,29,30,31].

Our work also serves as an illustration of how differences in the distributions of patients associated with race/ethnicity and insurance can occur at the area or facility level and how these data capture different types of information and serve different purposes. An area can be identified through the uneven distribution of its populations between subunits (such as through the Dissimilarity Index) [44,71], the low likelihood of interaction between populations of different groups (such as through the Isolation Index) [43], the extent of concentrations of high-privilege and low-privilege groups (such as using the Index of Concentration at the Extremes—race and income) [39,72,73], or the extent to which a subunit’s population composition diverges from that of the larger area (Divergence Index) [74,75,76]. A facility can be identified as disproportionately serving a certain group, whether relative to a national sample using a threshold measure (top decile), relative to a local healthcare market using a threshold measure (2×HRR), relative to a larger group using standard deviation (Shannon Diversity Index) [77], or relative to a local healthcare market, using a continuous measure (LHS index) [78].

Acknowledging an area and then individual facilities as disproportionate by any of these facets can prompt further examination of dynamic systems that concentrate resources and positive outcomes and inform interventions that reduce disproportion to help improve health outcomes for all. Hospitals are often even more or less disproportionate than can be explained by local residential disproportions [6,7,78,79]. Interventions to address patient sorting, such as through patient awareness, pre-hospital transport systems [80], emergency department transfers [81,82], interfacility transfers [83], and payment reform [84], can help mitigate the concentration of Black, Hispanic, minority, and dual-eligible patients in poorly resourced and lower-quality hospitals. Policies that aim to improve health outcomes by ensuring more even distributions of patients and resources can first identify areas with the greatest disproportions, followed by the facilities with the greatest disproportions, as targets for intervention.

**Figure 3:**
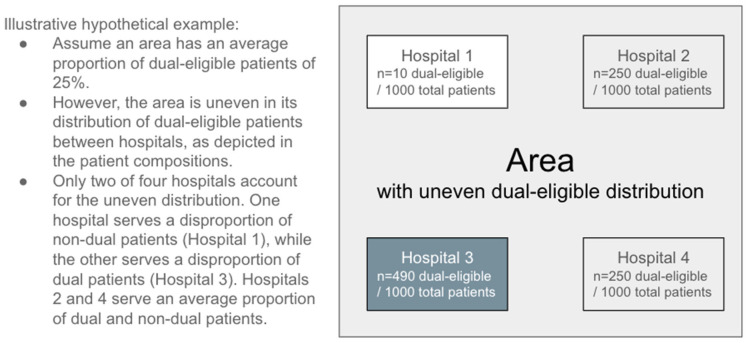
Illustrative hypothetical example of an area with an uneven distribution of dual-eligible patients due to disproportion in a subset of hospitals. Shades of gray indicate proportions of dual-eligible patients.

This stratified approach may be especially appropriate given that prior work has noted that measures of disproportion using evenness (DI) and measures using thresholds have inconsistent overlap, with correlation coefficients ranging from 0.062 [85] to 0.26 [86]. Our recent work found the correlation coefficients between DI and racial/ethnic compositions of HRRs to be low at 0.12–0.34 [6]. This low correlation is not surprising. An area may be considered uneven due to a subset of hospitals serving significantly disproportionate numbers of certain groups, which we have illustrated in Figure 3.

Recent work by others has also called for improved methods to measure how hospitals disproportionately serve patients belonging to certain groups. Akre et al. proposed an LHS index, which is defined as the difference between the racial composition of a hospital’s admissions and the racial composition of a hospital’s market [78]. The advantages of this approach are the specification of the direction of disproportion (i.e., positive values could indicate serving Black populations disproportionately, while negative values could indicate serving White populations disproportionately) and the increased granularity with a continuous measure. It should be considered for appropriate research questions. That being said, in practice, threshold measures resulting in a dichotomous, rather than continuous, measure of disproportion may still have utility. While threshold measures have been critiqued for relying on ad hoc definitions or arbitrary thresholds set by researchers [53], threshold measures are accessible and intuitive measures of disproportion at the facility level.

Our study’s findings and conclusions are subject to several limitations. Our data were limited to FFS Medicare beneficiaries, which in 2023, represented 49% of Medicare beneficiaries [87]. Future work should examine disproportion measures for hospitals using or including Medicare Advantage beneficiaries. For spatial characterization of healthcare markets, we used the widely accepted Dartmouth Atlas [88,89]. However, these boundaries are based on 1992–1993 data and have been critiqued for being too large to reflect local variation [90]. Contemporary HRRs, which are more granular [88], and HRRs based on state/county [89] have been proposed but are not yet widely in use. Moreover, methods used to crosswalk ZIP HRRs can also introduce errors, as ZIP boundaries can change over time. While the Atlas will not continue to update data [91], our approach of using two times the local geographic mean can be applied to other units, such as states, metropolitan areas, or other areas of interest.

Other limitations include the fact that the Dissimilarity Index does not account for relative group size and thus can overstate disproportions in smaller subunits and understate disproportions in larger subunits [63,64]. Relative group size is accounted for in the Atkinson Index [92]; however, we chose not to use the Atkinson Index given the additional complexity of its calculation and given that the DI is well-established and widely used as a measure of the evenness of populations across facilities. Our study also did not examine disproportionate populations within hospitals, which has been addressed by other scholars [93,94].

Future research can use the combined measure described in our paper to study disparities in health access and outcomes and how these disparities have changed over time. The combined measure can also be used to examine receipt of care in hospitals, with specification of the relative contributions of residential, healthcare market, hospital, and patient characteristics to receipt of care in disproportionate hospitals. The close relationship between minority and dual-eligible status also warrants more attention. Future research can use longitudinal methods and granular data on the uneven distribution of resources among White and minority patients; hospital patient composition by race, ethnicity, and insurance status; hospital reimbursement and resources; and patient outcomes to disentangle and further specify the mechanisms of this close relationship.

## 5. Conclusions

Differences in the composition of hospitalized patients by race, ethnicity, and insurance can occur at the area and facility levels. These differences are associated with disparate resources and outcomes, suggesting interventions to reduce disproportions between certain groups in poorly resourced hospitals can improve outcomes for all. Our study uses a novel combined measure to identify hospitals disproportionately serving different groups. By using disproportion associated with multiple characteristics and disproportion at both a national and regional level, this measure identifies a more comprehensive group of hospitals as disproportionate, while still detecting known differences in hospital quality with moderate statistical significance.

## Figures and Tables

**Table 1:** Number and percent of hospitals (total *n* = 2420) considered disproportionate by top decile, 2×HRR, and combined measures.

	Top Decile or 2×HRR (Combined Measure, A + B + C)	Top Decile Only (Subgroup A)	Both Top Decile and 2×HRR (Subgroup B)	2×HRR Only (Subgroup C)
Black	348 (14.4%)	146 (6.0%)	96 (4.0%)	106 (4.4%)
Hispanic	342 (14.1%)	183 (7.6%)	59 (2.4%)	100 (4.1%)
Minority	637 (26.3%)	368 (15.2%) ^a^	177 (7.3%) ^a^	215 (8.9%)
Dual-eligible	274 (11.3%)	196 (8.1%)	46 (1.9%)	32 (1.3%)
Black, Hispanic, minority, or dual-eligible	680 (28.1%)	423 (17.5%)	168 (6.9%)	238 (9.8%)

^a^ The sum of these two proportions would typically be expected to be 10% for the top decile; however, for minority race/ethnicity, it exceeds 10% due to this being an inclusive measure for hospitals that meet the top decile for Black, Hispanic, *or* other non-White minority groups.

**Table 2:** Mean hospital and area characteristics for hospitals identified by top decile (*n* = 242) measure and incrementally by 2×HRR only measure.

	Black			Hispanic			Minority			Dual		
	Top Decile (*n* = 242)	2×HRR Only (*n* = 106)	*p*-Value	Top Decile (*n* = 242)	2×HRR Only (*n* = 100)	*p*-Value	Top Decile (*n* = 480) ^a^	2×HRR Only (*n* = 157)	*p*-Value	Top Decile (*n* = 242)	2×HRR Only (*n* = 32)	*p*-Value
**Discharges per year** Median (IQR)	1334 (744–2198)	937 (510–1889)	0.001 **	1054 (637–1922)	579 (335–1264)	<0.001 ***	1212 (680–2120)	722 (371–1739)	<0.001 ***	1001 (637–1617)	588 (410–895)	0.001 **
**Number of hospitals in HRR** Median (IQR)	13.0 (9.0–22.0)	14.0 (6.0–23.0)	0.212	16.0 (7.0–24.0)	14.5 (7.0–23.5)	0.188	13.0 (8.0–23.0)	14.0 (6.0–23.0)	0.079	19.0 (10.0–25.0)	21.0 (9.0–38.0)	0.815
**Corresponding** **DI ^b^ of HRR** Mean (SD)	0.281 (0.124)	0.297 (0.084)	0.246	0.228 (0.094)	0.207 (0.068)	0.042 *	0.246 (0.100)	0.208 (0.087)	<0.001 ***	0.265 (0.109)	0.181 (0.055)	<0.001 ***
**CMS star quality rating** Mean (SD)	2.45 (1.09)	3.01 (1.05)	<0.001 ***	2.52 (1.11)	2.83 (1.02)	0.016 *	2.57 (1.11)	2.94 (1.03)	<0.001 ***	2.30 (1.09)	2.53 (1.08)	0.262
**Percent rural** (Number rural hospitals/total)	11.2% (27/242)	17.0% (18/106)	0.136	4.1% (10/242)	42.0% (42/100)	<0.001 ***	8.8% (42/480)	36.3% (57/157)	<0.001 ***	8.3% (20/242)	43.8% (14/32)	<0.001 ***
**Census region** (Column percent)	NE- 37 (15.3%) MW- 43 (17.8%) S- 145 (59.9%) W- 17 (7.0%)	NE- 24 (22.6%) MW- 28 (26.4%) S- 12 (11.3%) W- 42 (39.6%)	<0.01 ~~	NE- 21 (8.7%) MW- 9 (3.7%) S- 66 (27.3%) W- 146 (60.3%)	NE- 28 (28.0%) MW- 31 (31.0%) S- 39 (39.0%) W- 2 (2.0%)	<0.01 ~~	NE- 48 (10.0%) MW- 47 (9.8%) S- 207 (43.1%) W- 178 (37.1%)	NE- 39 (24.8%) MW- 53 (33.8%) S- 39 (24.8%) W- 26 (16.6%)	<0.01 ~~	NE- 51 (21.1%) MW- 28 (11.6%) S- 40 (16.5%) W- 123 (50.8%)	NE- 2 (6.3%) MW- 3 (9.4%) S- 23 (71.9%) W- 4 (12.5%)	<0.01 ~~

Note: *** *p* < 0.001, ** *p* < 0.01, * *p* < 0.05. DI, Dissimilarity Index; HRR, hospital referral region; NE, Northeast; MW, Midwest; S, South; W, West. ~~ *p* < 0.01 for chi-squared test. ^a^ Top decile would typically be considered 10% of the total sample of 2420 hospitals; however, for minority race/ethnicity, it exceeds 10% due to this being an inclusive measure for hospitals that meet the top decile for Black, Hispanic, or other non-White minority groups. ^b^ Corresponding DI refers to the Dissimilarity Index of the corresponding group, i.e., for Black-serving hospitals, the mean DI-Black; for Hispanic-serving hospitals, the mean DI-Hispanic, etc.

**Table 3:** Mean hospital and area characteristics for Black-, Hispanic-, minority-, and dual-eligible-serving hospitals versus not using the **top decile measure**.

	Black			Hispanic			Minority			Dual		
	Black-Serving (*n* = 242)	Not Black-Serving (*n* = 2178)	*p*-Value	Hispanic- Serving (*n* = 242)	Not Hispanic-Serving (*n* = 2178)	*p*-Value	Minority- Serving (*n* = 480) ^a^	Not Minority-Serving (*n* = 1940)	*p*-Value	Dual-Serving (*n* = 242)	Not Dual-Serving (*n* = 2178)	*p*-Value
**Discharges per year** Median (IQR)	1334 (744–2198)	1524 (779–2799)	0.026 *	1054 (637–1922)	1566 (798–2856)	<0.001 ***	1212 (680–2120)	1610 (803–2914)	<0.001 ***	1001 (637–1617)	1606 (801–2878)	<0.001 ***
**Number of hospitals in HRR** Median (IQR)	14.0 (9.0–24.0)	12.0 (6.0–23.0)	<0.001 ***	18.0 (9.0–26.0)	12.0 (6.0 = 23.0)	<0.001 ***	15.0 (9.0–24.0)	12.0 (6.0–22.0)	<0.001 ***	20.0 (10.0–28.0)	12.0 (6.0–22.0)	<0.001 ***
**Corresponding** **DI ^b^ of HRR** Mean (SD)	0.281 (0.104)	0.246 (0.240–0.249)	<0.001 ***	0.228 (0.094)	0.165 (0.073)	<0.001 ***	0.246 (0.100)	0.185 (0.091)	<0.001 ***	0.265 (0.109)	0.146 (0.071)	<0.001 ***
**CMS star quality rating** Mean (SD) ^c^	2.45 (1.09)	3.18 (1.15)	<0.001 ***	2.52 (1.15)	3.17 (1.11)	<0.001 ***	2.57 (1.11)	3.24 (1.14)	<0.001 ***	2.30 (1.09)	3.19 (1.14)	<0.001 ***
**Percent rural** (Number rural hospitals/total)	11.2% (27/242)	23.7% (515/2178)	<0.001 ***	4.1% (10/242)	24.4% (532/2178)	<0.001 ***	8.8% (42/480)	25.8% (500/1940)	<0.001 ***	8.3% (20/242)	24.0% (522/2178)	<0.001 ***
**Census region** (Column percent)	NE- 37 (15.3%) MW- 43 (17.8%) S- 145 (59.9%) W- 17 (7.0%)	NE- 367 (16.9%) MW- 534 (24.5%) S- 800 (36.7%) W-477 (21.9%)	<0.01 ~~	NE- 21 (8.7%) MW- 9 (3.7%) S- 66 (27.3%) W- 146 (60.3%)	NE- 383 (17.6%) MW- 568 (26.1%) S- 879 (40.4%) W-348 (16.0%)	<0.01 ~~	NE- 48 (10.0%) MW- 47 (9.8%) S- 207 (43.1%) W- 178 (37.1%)	NE- 356 (18.4%) MW- 530 (27.3%) S- 738 (38.0%) W-316 (16.3%)	<0.01 ~~	NE- 51 (21.1%) MW- 28 (11.6%) S- 40 (16.5%) W- 123 (50.8%)	NE- 353 (16.2%) MW- 549 (25.2%) S- 905 (41.6%) W-371 (17.0%)	<0.01 ~~

Note: *** *p* < 0.001, * *p* < 0.005. DI, Dissimilarity Index; HRR, hospital referral region; NE, Northeast; MW, Midwest; S, South; W, West. ~~ *p* < 0.01 for chi-squared test. ^a^ Top decile would typically be considered 10% of the total sample of 2420 hospitals; however, for minority race/ethnicity, it exceeds 10% due to this being an inclusive measure for hospitals that meet the top decile for Black, Hispanic, or other non-White minority groups. ^b^ Corresponding DI refers to the Dissimilarity Index of the corresponding group, i.e., for Black-serving hospitals, the mean DI-Black; for Hispanic-serving hospitals, the mean DI-Hispanic, etc. ^c^ SMDs of −0.65, −0.57, −0.59, and −0.80 for Black-serving, Hispanic-serving, minority-serving, and dual-serving hospitals, respectively.

**Table 4:** Mean hospital and area characteristics for Black-, Hispanic-, minority-, and dual-eligible-serving hospitals versus not using the **combined measure** of top decile or 2×HRR.

	Black			Hispanic			Minority			Dual		
	Black-Serving (*n* = 348)	Not Black-Serving (*n* = 2072)	*p*-Value	Hispanic- Serving (*n* = 342)	Not Hispanic-Serving (*n* = 2078)	*p*-Value	Minority- Serving (*n* = 637) ^a^	Not Minority-Serving (*n* = 1783)	*p*-Value	Dual-Serving (*n* = 274)	Not Dual-Serving (*n* = 2146)	*p*-Value
**Discharges per year** Median (IQR)	1157 (657–2106)	1573 (796–2861)	<0.001 ***	888 (514–1682)	1634 (838–2919)	<0.001 ***	1083 (589–2061)	1691 (854–3067)	<0.001 ***	939 (589–1515)	1624 (811–2914)	<0.001 ***
**Number of hospitals in HRR** Median (IQR)	14.0 (8.0–24.0)	12.0 (6.0–23.0)	0.004 **	18.0 (8.0–26.0)	12.0 (6.0–22.0)	<0.001 ***	15.0 (8.0–24.0)	12.0 (6.0–22.0)	<0.001 ***	21.0 (10.0–32.0)	12.0 (6.0–22.0)	<0.001 ***
**Corresponding** **DI ^a^ of HRR** Mean (SD)	0.286 (0.113)	0.243 (0.105)	<0.001 ***	0.222 (0.088)	0.163 (0.073)	<0.001 ***	0.232 (0.099)	0.185 (0.091)	<0.001 ***	0.255 (0.108)	0.145 (0.071)	<0.001 ***
**CMS star quality rating** Mean (SD) ^b^	2.62 (1.10)	3.19 (1.16)	<0.001 ***	2.61 (1.09)	3.19 (1.16)	<0.001 ***	2.67 (1.10)	3.26 (1.15)	<0.001 ***	2.33 (1.09)	3.20 (1.14)	<0.001 ***
**Percent rural** (Number rural hospitals/total)	12.9% (45/348)	24.0% (497/2072)	<0.001 ***	15.2% (52/342)	23.6% (490/2078)	<0.001 ***	15.5% (99/637)	24.9% (443/1783)	<0.001 ***	12.4% (34/274)	23.7% (508/2146)	<0.001 ***
**Census region** (Column percent)	NE- 61 (17.5%) MW- 71 (20.4%) S- 157 (45.1%) W- 59 (17.0%)	NE- 343 (16.6%) MW- 506 (24.4%) S- 788 (38.0%) W- 435 (21.0%)	<0.01 ~~	NE- 49 (14.3%) MW- 40 (11.7%) S- 105 (30.7%) W- 148 (43.3%)	NE- 355 (17.1%) MW- 537 (25.8%) S- 840 (40.4%) W- 346 (16.7%)	<0.01 ~~	NE- 87 (13.7%) MW- 100 (15.7%) S- 246 (38.6%) W- 204 (32.0%)	NE- 317 (17.8%) MW- 477 (26.8%) S- 699 (39.2%) W- 290 (16.3%)	<0.01 ~~	NE- 53 (19.3%) MW- 31 (11.3%) S- 63 (23.0%) W- 127 (46.4%)	NE- 351 (16.4%) MW- 546 (25.4%) S- 882 (41.1%) W- 367 (17.1%)	<0.01 ~~

Note: *** *p* < 0.001, ** *p* < 0.01. DI, Dissimilarity Index; HRR, hospital referral region; NE, Northeast; MW, Midwest; S, South; W, West. ~~ *p* < 0.01 for chi-squared test. ^a^ Corresponding DI refers to the Dissimilarity Index of the corresponding group, i.e., for Black-serving hospitals, the mean DI-Black; for Hispanic-serving hospitals, mean DI-Hispanic, etc. ^b^ SMDs of −0.50, −0.51, −0.52, and −0.79 for Black-serving, Hispanic-serving, minority-serving, and dual-serving hospitals, respectively.

## Data Availability

The raw data supporting the conclusions of this article will be made available by the authors on request.

## Abbreviations

The following abbreviations are used in this manuscript:

BMC	Boston Medical Center
CI	Confidence interval
CMS	Centers for Medicare and Medicaid Services
DI	Dissimilarity Index
FFS	Fee-for-service
HRR	Hospital referral region
IQR	Interquartile range
RUCA	Rural–urban commuting area
SES	Socioeconomic status
SMD	Standardized mean difference

## Appendix A

**Table A1:** Number of hospitals disproportionately serving Black, Hispanic, minority, and dual-eligible beneficiaries by varying multiples of HRR means.

Measure of Disproportion	Black		Hispanic		Non-White (Minority)		Dual		Black, Hispanic, Minority, or Dual-Eligible ^a^
	Mean Threshold of Black Discharges (±SD)	Black-Serving Hospitals: Number (%)	Mean Threshold of Hispanic Discharges (±SD)	Hispanic-Serving Hospitals: Number (%)	Mean Threshold of Non-White Discharges (±SD)	Minority-Serving Hospitals: Number (%)	Mean Threshold of Dual Discharges (±SD)	Dual-Serving Hospitals: Number (%)	Hospitals: Number (%)
1.5×HRR average	21.93% (±20%)	420 (17.4%)	14.91% (±19%)	366 (15.1%)	33.73% (±25%)	718 (29.7%)	47.30% (±20%)	305 (12.6%)	794 (32.8%)
2×HRR average	27.62% (±23%)	202 (8.4%)	18.36% (±22%)	159 (6.6%)	39.83% (±28%)	334 (13.8%)	52.82% (±16%)	78 (3.2%)	356 (14.7%)
2.5×HRR average	35.09% (±25%)	104 (4.3%)	21.88% (±24%)	75 (3.1%)	46.54% (±28%)	167 (6.9%)	62.35% (±15%)	21 (0.9%)	171 (7.1%)

^a^ Mean threshold of Black, Hispanic, minority, or dual-serving discharges for hospitals could not be calculated due to not having patient-level data on the number of unique patients in this group, as these characteristics can overlap.

**Table A2:** Mean CMS star quality rating and standardized mean difference (SMD) between disproportionate and non-disproportionate hospitals when using 1.5×HRR threshold.

	*t*-Tests				
	Disproportionate Hospitals	Non-Disproportionate Hospitals	*p*-Value	Difference in Means	SMD
Black	2.75 (*n* = 420)	3.18 (*n* = 2000)	<0.001	0.43	0.37
Hispanic	2.75 (*n* = 366)	3.17 (*n* = 2054)	<0.001	0.42	0.36
Minority	2.78 (*n* = 718)	3.24 (*n* = 1702)	<0.001	0.46	0.39
Dual-eligible	2.39 (*n* = 305)	3.21 (*n* = 2115)	<0.001	0.82	0.71

**Table A3:** Minority-serving hospitals that are not Black- or Hispanic-serving hospitals (*n* = 35).

HRR #	HRR City	Hospital Identifier (CCN)	Hospital Name	Hospital City	State	Hospital Percentage API	HRR Percentage API	Hospital Percentage AI/AN	HRR Percentage AI/AN	Hospital Percentage Minority	HRR Percentage Minority
10	Anchorage	20026	Alaska Native Medical Center	Anchorage	AK	0.36	4.46	78.82	16.68	90.76	31.23
12	Phoenix	30023	Flagstaff Medical Center	Flagstaff	AZ	0.53	1.21	28.50	4.64	38.17	18.77
23	Orange County	50230	Garden Grove Hospital & Medical Center	Garden Grove	CA	67.42	20.80	0.60	0.27	82.82	36.64
23	Orange County	50570	UCI Health Fountain Valley	Fountain Valley	CA	56.49	20.80	0.24	0.27	73.50	36.64
23	Orange County	50678	Memorial Care Orange Coast Medical Center	Fountain Valley	CA	49.54	20.80	0.23	0.27	60.59	36.64
56	Los Angeles	50737	Garfield Medical Center	Monterey Park	CA	73.73	12.41	0.39	0.29	92.76	48.34
56	Los Angeles	50132	San Gabriel Valley Medical Center	San Gabriel	CA	62.26	12.41	0.34	0.29	84.57	48.34
56	Los Angeles	50353	Providence Little Company of Mary Med Ctr Torrance	Torrance	CA	13.97	12.41	0.21	0.29	50.21	48.34
65	Alameda County	50195	Washington Hospital	Fremont	CA	32.18	19.02	0.20	0.32	57.59	51.70
65	Alameda County	50211	Alameda Hospital	Alameda	CA	18.29	19.02	0.71	0.32	55.57	51.70
65	Alameda County	50488	Eden Medical Center	Castro Valley	CA	17.52	19.02	0.25	0.32	49.82	51.70
81	San Francisco	50407	Chinese Hospital	San Francisco	CA	95.51	17.18	1.60	0.44	98.40	40.15
81	San Francisco	50076	Kaiser Foundation Hospital—San Francisco	San Francisco	CA	25.06	17.18	1.28	0.44	61.89	40.15
81	San Francisco	50055	California Pacific Medical Center—Mission Bernal	San Francisco	CA	19.57	17.18	0.39	0.44	51.66	40.15
85	San Mateo County	50289	AHMC Seton Medical Center	Daly City	CA	32.02	15.57	0.79	0.25	62.30	34.70
150	Honolulu	120007	Kuakini Medical Center	Honolulu	HI	69.57	46.35	0.70	0.39	88.08	57.67
150	Honolulu	120026	Pali Momi Medical Center	Aiea	HI	60.49	46.35	0.35	0.39	84.39	57.67
150	Honolulu	120001	The Queens Medical Center	Honolulu	HI	50.30	46.35	0.10	0.39	71.77	57.67
150	Honolulu	120011	Kaiser Foundation Hospital	Honolulu	HI	42.27	46.35	0.98	0.39	70.06	57.67
150	Honolulu	120022	Straub Clinic And Hospital	Honolulu	HI	47.83	46.35	0.30	0.39	66.25	57.67
150	Honolulu	120006	Adventist Health Castle	Kailua	HI	43.39	46.35	0.51	0.39	64.74	57.67
150	Honolulu	120005	Hilo Medical Center	Hilo	HI	39.89	46.35	0.39	0.39	57.70	57.67
150	Honolulu	120014	Wilcox Memorial Hospital	Lihue	HI	32.87	46.35	0.78	0.39	49.77	57.67
227	Boston	220116	Tufts Medical Center	Boston	MA	5.80	1.60	0.16	0.15	21.77	10.41
250	Duluth	240019	Essentia Health Duluth	Duluth	MN	0.83	0.25	4.67	1.65	12.00	5.30
267	Joplin	370113	Integris Grove Hospital	Grove	MO	1.38	0.54	13.77	2.97	15.70	7.41
281	Lebanon	300003	Mary Hitchcock Memorial Hospital	Lebanon	NH	0.39	0.45	0.10	0.22	5.70	2.81
293	Albuquerque	320061	Gallup Indian Medical Center	Gallup	NM	1.34	0.86	93.30	16.41	98.66	43.22
301	East Long Island	330055	New York-Presbyterian/Queens	Flushing	NY	18.57	4.31	0.12	0.18	49.26	22.20
322	Fargo/Moorhead, MN	240100	Sanford Bemidji Medical Center	Bemidji	ND	0.48	0.43	16.78	4.48	20.44	9.02
323	Grand Forks	350019	Altru Hospital	Grand Forks	ND	0.19	0.19	0.19	0.19	9.96	4.96
329	Columbus	360085	Ohio State University Hospitals	Columbus	OH	0.87	0.79	0.08	0.29	18.70	9.26
339	Oklahoma City	370180	Chickasaw Nation Medical Center	Ada	OK	1.67	0.89	79.33	4.96	83.67	15.19
340	Tulsa	370171	W W Hastings Indian Hospital	Tahlequah	OK	0.95	0.74	81.82	10.55	86.74	19.21
457	Casper	530008	Sagewest Health Care	Riverton	WY	1.02	0.34	20.08	3.55	23.98	10.13

**Table A4:** SMDs of CMS star quality ratings for disproportionate versus non-disproportionate hospitals for combined measure (A + B + C) compared to top decile (subgroup A + B).

	Top Decile or 2×HRR (Group A + B + C)	SMD (95% CI)	Top Decile (Subgroup A + B)	SMD (95% CI)
Black	348 (14.4%)	−0.50 (0.37–0.60)	242 (10.0%)	−0.65 (0.50–0.76)
Hispanic	342 (14.1%)	−0.51 (0.39–0.62)	242 (10.0%)	−0.57 (0.43–0.70)
Minority	637 (26.3%)	−0.52 (0.42–0.61)	480 (19.8%) ^a^	−0.59 (0.48–0.69)
Dual-eligible	274 (11.3%)	−0.79 (0.65–0.90)	242 (10.0%)	−0.80 (0.65–0.92)
Black, Hispanic, minority, or dual-eligible	680 (28.1%)	−0.54 (0.45–0.63)	522 (21.6%)	−0.62 (0.52–0.71)

^a^ Proportion exceeds 10% due to being an inclusive measure for hospitals that meet top decile for Black, Hispanic, *or* other non-White minority groups.

**Table A5:** Mean CMS star quality ratings and SMDs for disproportionate and non-disproportionate hospitals using top decile, combined, and 2×HRR measures.

	Top Decile			Both Top Decile and 2×HRR			2×HRR		
	Disproportionate Hospital	Non-Disproportionate Hospital	SMD	Disproportionate Hospital	Non-Disproportionate Hospital	SMD	Disproportionate Hospital	Non-Disproportionate Hospital	SMD
**Black**	2.46	3.18	0.64	2.62	3.19	0.50	2.64	3.15	0.44
**Hispanic**	2.52	3.17	0.57	2.61	3.19	0.51	2.66	3.14	0.43
**Minority**	2.57	3.24	0.59	2.67	3.26	0.52	2.70	3.17	0.42
**Dual**	2.30	3.19	0.80	2.33	3.20	0.79	2.32	3.13	0.74
